# Albumin Thiolation and Oxidative Stress Status in Patients with Aortic Valve Stenosis

**DOI:** 10.3390/biom13121713

**Published:** 2023-11-28

**Authors:** Carlo Savini, Elena Tenti, Elisa Mikus, Sonia Eligini, Marco Munno, Anna Gaspardo, Erica Gianazza, Arianna Greco, Stefania Ghilardi, Giancarlo Aldini, Elena Tremoli, Cristina Banfi

**Affiliations:** 1 GVM Care and Research, Maria Cecilia Hospital, 48033 Cotignola, Italy; csavini@gvmnet.it (C.S.); etenti@gvmnet.it (E.T.); emikus@gvmnet.it (E.M.); etremoli@gvmnet.it (E.T.); 2 Dipartimento di Scienze Mediche e Chirurgiche, Alma Mater Studiorum, Università di Bologna, 40126 Bologna, Italy; 3 Unit of Functional Proteomics, Metabolomics, and Network Analysis, Centro Cardiologico Monzino IRCCS, 20138 Milano, Italy; sonia.eligini@cardiologicomonzino.it (S.E.); marco.munno@cardiologicomonzino.it (M.M.); anna.gaspardo@cardiologicomonzino.it (A.G.); erica.gianazza@cardiologicomonzino.it (E.G.); arianna.greco@cardiologicomonzino.it (A.G.); stefania.ghilardi@cardiologicomonzino.it (S.G.); 4 Department of Pharmaceutical Sciences, University of Milan, 20133 Milano, Italy; giancarlo.aldini@unimi.it

**Keywords:** albumin, oxidative stress, valve replacement, free sulfhydryl groups, serum antioxidant activity

## Abstract

Recent evidence indicates that reactive oxygen species play an important causative role in the onset and progression of valvular diseases. Here, we analyzed the oxidative modifications of albumin (HSA) occurring on Cysteine 34 and the antioxidant capacity of the serum in 44 patients with severe aortic stenosis (36 patients underwent aortic valve replacement and 8 underwent a second aortic valve substitution due to a degenerated bioprosthetic valve), and in 10 healthy donors (controls). Before surgical intervention, patients showed an increase in the oxidized form of albumin (HSA-Cys), a decrease in the native reduced form (HSA-SH), and a significant reduction in serum free sulfhydryl groups and in the total serum antioxidant activity. Patients undergoing a second valve replacement showed levels of HSA-Cys, free sulfhydryl groups, and total antioxidant activity similar to those of controls. In vitro incubation of whole blood with aspirin (ASA) significantly increased the free sulfhydryl groups, suggesting that the in vivo treatment with ASA may contribute to reducing oxidative stress. We also found that N-acetylcysteine and its amide derivative were able to regenerate HSA-SH. In conclusion, the systemic oxidative stress reflected by high levels of HSA-Cys is increased in patients with aortic valve stenosis. Thiol–disulfide breaking agents regenerate HSA-SH, thus paving the way to the use these compounds to mitigate the oxidative stress occurring in the disease.

## 1. Introduction

Aortic valve stenosis is a serious and complex condition that affects millions of patients worldwide and is associated with high morbidity and mortality [1]. Its prevalence is increasing as the population ages, with an incidence of more than 10% in individuals older than 65 years of age in US and European populations [2,3]. 

Due to the absence of pharmacological therapies available for preventing, treating, or slowing the development of aortic valve stenosis, understanding the mechanisms underlying the onset and progression of the disease is crucial for identifying novel therapeutic targets. Indeed, the treatment of severe valvular disease is based on surgical intervention (TAVR, transcatheter aortic valve replacement, or TAVI, transcatheter aortic valve implantation) in which, with a minimally invasive procedure, a dysfunctional valve is replaced by an artificial valve, such as a mechanical valve or bioprosthetic valve [4]; currently, around 300,000 artificial valves are implanted every year, a number that is set to increase further [5].

Recent findings have highlighted an important causative role for reactive oxygen species (ROS)-mediated oxidative stress in the onset and progression of valvular disease, which can contribute to the differentiation of valve interstitial cells into myofibroblasts and osteoblasts [6]. Indeed, oxidative stress, a condition derived from an imbalance between ROS production and ROS degradation, is involved in several pathological processes, such as inflammation, lipid infiltration, and calcification, all aspects that participate in cardiovascular disease, including aortic valve disease [7]. In addition, ROS induce the oxidation of several molecules, redox gene overexpression, and DNA fragmentation, all of which contribute to myocardial cell damage [8]. Proteins are important targets of ROS, which can irreversibly damage them with deleterious consequences on their function [9].

In addition to the irreversible oxidative damage, the protein function can be altered by the reversible oxidation of the thiol group of the cysteine residues, so much so that proteins containing thiol groups may be potential biomarkers of oxidative stress [10]. Among these, albumin (HSA), through the free sulfhydryl group present on cysteine residue in position 34 (Cys34), exerts antioxidant activity and protects from systemic oxidative stress [11,12,13]. Therefore, HSA represents the major and predominant antioxidant in plasma whose relative contribution was reported to range from 40% to 70% of the total antioxidant capacity. Cys34 plays the most important role in the antioxidant activity of HSA against the tested oxidant species, namely O2^−●^, NO, HO^●^, HOCl, and H_2_O_2_. Furthermore, in vitro, HSA is an effective detoxifying nucleophilic agent of α-β unsaturated aldehydes generated from lipid peroxidation, including 4-hydroxy-trans-2-nonenal and other reactive carbonyls, such as glyoxal and methylglyoxal [14]. Considering its abundance, HSA and its corresponding oxidized forms represent valid and robust biomarkers of systemic oxidative stress and are predictors of the onset and development of some oxidation-based diseases. In healthy subjects, 70–80% of Cys34 present in HSA is in the free sulfhydryl form (mercaptoalbumin, HSA-SH), with the rest being oxidized to higher oxidation states where the thiol is modified in a reversible (non-mercaptolbumin 1, HNA-1) or irreversible (HNA-2) manner [15,16]. In HNA-1, accounting for 20–30%, the reduced thiol group (–SH) forms a disulfide bond with low molecular weight thiols, such as cysteine, cysteinylglycine, homocysteine, and glutathione, whilst, in HNA-2, which accounts for approximately 2–5%, the oxidation of SH is responsible for forming reaction products, such as sulfinic and sulfonic acid derivatives. Based on the principle that HSA-SH is primarily converted to cysteinylated HSA (HSA-Cys) to maintain redox balance, the HSA proteoforms may provide a useful measure of the body’s ability to maintain redox balance [17]. Accordingly, we have recently shown that patients affected by heart failure, a pathological condition characterized by inflammation and oxidative stress, had elevated plasma levels of HSA-Cys, the oxidized form of HSA, with a simultaneous decrease in the native reduced form of HSA (HSA-SH) [18]. In vitro experiments also showed that HSA-Cys does not protect the cells from the cytotoxic effect induced by H_2_O_2_, indicating that oxidation impairs the protective function of HSA [18]. Furthermore, the redox state of HSA directly influences platelet functions [19].

Because recent evidence has emerged of an important causative role for ROS-mediated oxidative stress in the pathophysiology of valve diseases [6], in this study, we evaluated the HSA oxidative status in patients with aortic valve stenosis undergoing aortic valve replacement, and in patients who have undergone a first replacement and then require a second valve replacement due to a degenerated bioprosthetic valve, compared to healthy subjects. Furthermore, since patients requiring a second surgery are in pharmacological treatment with aspirin (ASA), we tested in vitro the capability of ASA and thiol–disulfide breaking agents to affect the levels of the free sulfhydryl groups.

## 2. Materials and Methods

### 2.1. Study Population

Forty-four patients undergoing valve replacement for calcific severe aortic stenosis at Maria Cecilia Hospital, Cotignola (RA), Italy, were enrolled between September 2018 and May 2022. Of these, 36 patients had an aortic valve replacement and 8 underwent a second aortic valve substitution by surgical aortic valve replacement. Patients must have met all of the following inclusion criteria: age > 65, calcific severe aortic valve stenosis, hospitalization to perform aortic valve replacement or redo aortic valve replacement for a degenerated bioprosthetic valve, and patient able to understand the trial and to sign the informed consent. The exclusion criteria included the following: rheumatic valve disease, infective endocarditis, end-stage kidney disease, hematological disorders, severe cognitive impairment (Short Portable Mental Status Questionnaire, SPMSQ < 4), life expectancy < 12 months for non-cardiac causes, hepatic dysfunction (bilirubin > 20 mmol/L, prothrombin > 2.0 ratio), severe renal failure (glomerular filtration rate < 30 mL/min/1.73 m^2^), and bioprosthetic degeneration caused by endocarditis. The functional data (left ventricular ejection fraction and aortic gradient) were assessed 24 h before the surgical procedure.

In comparison, 10 healthy donors (controls) with neither a history of cardiovascular disease nor inflammatory disorders and not taking cardiovascular therapy were recruited as the control group. A blood sample was taken before surgery; serum was obtained from blood clotted for 2 h at 37 °C and subsequently centrifuged at 1500× *g* for 15 min. Serum samples were stored at −80 °C until analysis. The study was approved by the institutional Ethics Committee, and it was performed in accordance with the principles of the Declaration of Helsinki. All the study participants provided written informed consent at the time of enrollment. 

### 2.2. Albumin Analysis by LC–Mass Spectrometry 

The composition of HSA proteoforms was assessed in serum samples obtained from patients and controls. Although several studies have evaluated the HSA redox status in serum [11], during the clotting process, the cell activation might affect the oxidative modification of HSA, leading to an overestimation of the results. To assess possible ex vivo oxidation in serum samples, we performed appropriate experiments comparing the HSA-Cys levels in serum and plasma obtained from the same subjects. As shown in Supplemental Figure S1, only a modest difference in HSA-Cys levels was detected between the two different compartments. We therefore analyzed the serum samples, because they were available from all subjects.

The HSA proteoforms were evaluated as previously described [20,21] with a direct infusion of the sample into a Xevo TQ-S micro triple quadrupole mass spectrometer coupled with the ACQUITY UPLC^®^ M-Class system (Waters Corporation, Milford, CT, USA). The samples centrifuged at 3000× *g* for 10 min at 4 °C were diluted (400-fold) with a solution of 30% acetonitrile and 0.1% formic acid. The samples were centrifuged at 14,000× *g* at 4 °C for 10 min, and then 2 µL were injected in a full loop mode at 5 µL/min. The spectra were acquired with the following parameters: positive ESI mode; mass range 1100–1350 *m*/*z*; MS scan in multi-channel acquisition with 1 s scan time; capillary voltage 3 kV; cone 90 V; desolvation temperature 350 °C; source temperature 150 °C. The data were processed for deconvolution with the MaxEnt 1TM function on the MassLynx software version V4.1 (Waters Corporation, Milford, CT, USA). Intensities of HSA proteoforms (native reduced HSA, HSA-SH; cysteinylated HSA, HSA-Cys; glycated HSA, HSA-Gly) were used to calculate the relative abundances as previously described [21]. Of note, the method does not us allow to measure the HNA2 proteoform, which represents a minor fraction of the HSA modifications (~2–5%). The mass of HSA was calculated by a deconvolution algorithm, which calculates the centroid of the mass distribution instead of the monoisotopic mass of the protein. For all the tested samples, a coefficient of variation of 7.0 ± 1.1% (mean ± SEM) was calculated for the percentages of HSA-Cys. 

### 2.3. Quantification of Free Sulfhydryl Groups in Serum from Patients and Controls

Free sulfhydryl groups were detected in serum samples obtained from all patients with aortic valve stenosis before valve replacement and healthy subjects using Ellman’s reagent, as previously described [22] with some modifications. Briefly, 25 µL of samples and 5 µL of Ellman’s reagent [4 mg 5,5′-dithio-bis-(2-nitrobenzoic acid)/mL in the reaction buffer (0.1 M sodium phosphate, pH 8.0 containing 1 mM EDTA)] were added to 250 µL of reaction buffer. Ellman’s reagent reacts with free sulfhydryl groups of proteins, generating the yellow-colored product 2-nitro-5-thiobenzoic acid. After 15 min at room temperature, the absorbance of each sample was measured at 412 nm. The concentration of free sulfhydryl groups was calculated using the molar extinction coefficient 14,150 M^−1^cm^−1^ after subtracting the background for each sample. 

### 2.4. In Vitro Treatment of Plasma with Aspirin

Free sulfhydryl groups were also detected in plasma samples obtained from healthy donors in vitro treated with or without ASA. For these experiments, 10 mL of venous blood were collected in Vacutainer^®^ tubes containing EDTA and incubated for 120 min at 37 °C with slow shaking in the presence or the absence of 100 µM ASA. After centrifugation at 1500× *g* for 15 min, plasma was collected, and free sulfhydryl groups were detected as described above. 

### 2.5. In Vitro Treatment of Serum from Patients with N-Acetylcysteine and N-Acetylcysteine Amide AD4/NACA 

Serum obtained from 6 patients with high levels of HSA-Cys before valve replacement was treated in vitro with vehicle (ammonium formate buffer pH 3.5), N-acetylcysteine (NAC, 0.6 mM), or N-acetylcysteine amide (AD4/NACA, 0.6 mM) for 1 h at 37 °C with stirring (300 rpm). HSA proteoforms (HSA-Cys, HSA-Gly, and HSA-SH) as well as the free sulfhydryl groups wre quantified as described above. 

### 2.6. Measurement of the Antioxidant Activity 

Serum obtained from 6 patients with high levels of HSA-Cys before valve replacement previously treated in vitro with vehicle (ammonium formate buffer pH 3.5) or N-acetylcysteine (NAC, 0.6 mM), or N-acetylcysteine amide (AD4/NACA, 0.6 mM) was treated with 2′,7′-dichlorodihydrofluorescin diacetate (DCFH-DA; Sigma Aldrich S.r.l., Milan, Italy) as a probe and 2,2′-Azobis (2-amidinopropane) dihydrochloride (AAPH, Sigma Aldrich S.r.l., Milan, Italy) as the radical generator. DCFH was obtained by basic hydrolysis of DCFH-DA, i.e., by mixing 500 µL of 1 mM DCFH-DA with 2 mL 0.01 M NaOH at 4 °C. After 20 min, the mixture was neutralized with 2 mL 0.01 M HCl. Samples (10 µL) were analyzed in triplicate in a 96-well black plate (Corning Incorporated Costar, Euroclone S.p.A., Milan, Italy) with the addition of 25 µL PBS, 1 µL AAPH (1 mM final concentration), and 5 µL DCFH (10 µM final concentration). The oxidation of DCFH to 2′,7′-dichlorofluorescin (DCF) was assessed by setting the excitation at λ 485 nm and emission at λ 535 nm, at 37 °C.

### 2.7. Statistical Analysis 

Continuous variables were reported as median and interquartile range (IQR) and compared with the Mann–Whitney test; categorical variables were reported as absolute number (*n*) and frequencies (%) and compared with the chi-squared test or Fisher exact test as appropriate. All analyses were performed with STATA 17.0 SE (StataCorp LLC, TX, USA); *p*-values < 0.05 were considered statistically significant.

Comparisons between the two groups were performed using paired Student’s *t*-tests. Comparisons among three groups were performed using ANOVA and Tukey’s or Dunnett’s post hoc tests, as indicated. Pearson correlation analysis was performed to test the relationship between serum HSA-Cys and free sulfhydryl groups.

All tests were 2-sided. A *p* ≤ 0.05 was considered statistically significant. 

## 3. Results

### 3.1. Characteristics of the Study Participants 

The study was performed on 44 patients with severe aortic valve stenosis, 36 of whom underwent aortic valve replacement and 8 of whom underwent a second aortic valve replacement. Participants had a median age of 74 years, with 21 women and 23 men; 88% had hypertension, 22% had diabetes, 70% had dyslipidemia, 9% had an oncological history, 16% had coronary artery disease, and 5% had previous coronary artery bypass graft (CABG). Moreover, 57% were in pharmacological treatment with statins and 14% were being treated with oral anticoagulants (Table 1).

### 3.2. Albumin Proteoforms in the Study Population 

**Figure 1:**
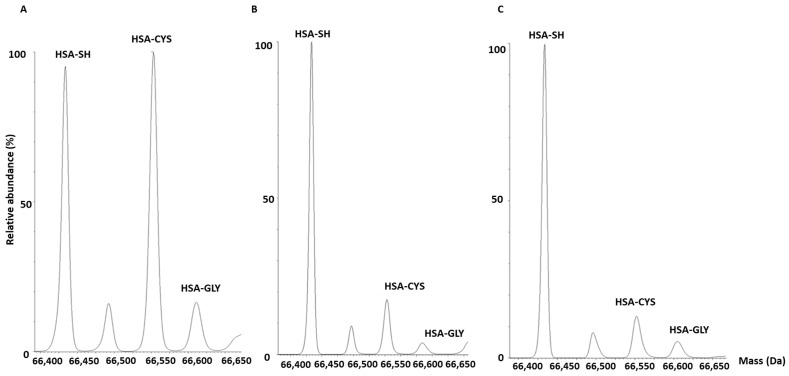
Representative chromatograms of different HSA proteoforms. Serum was obtained from (**A**) patients with aortic valve stenosis before valve replacement, (**B**) patients undergoing a second valve replacement, and (**C**) controls.

Total HSA and the different HSA proteoforms, namely HSA-Cys, HSA-Gly, and HSA-SH, were identified by the MS-based method in serum of patients with aortic valve stenosis undergoing aortic valve replacement, in patients who had already undergone valve replacement and required a second operation, and in controls. Representative chromatograms of the HSA isoforms in patients and controls are shown in Figure 1.

**Figure 2:**
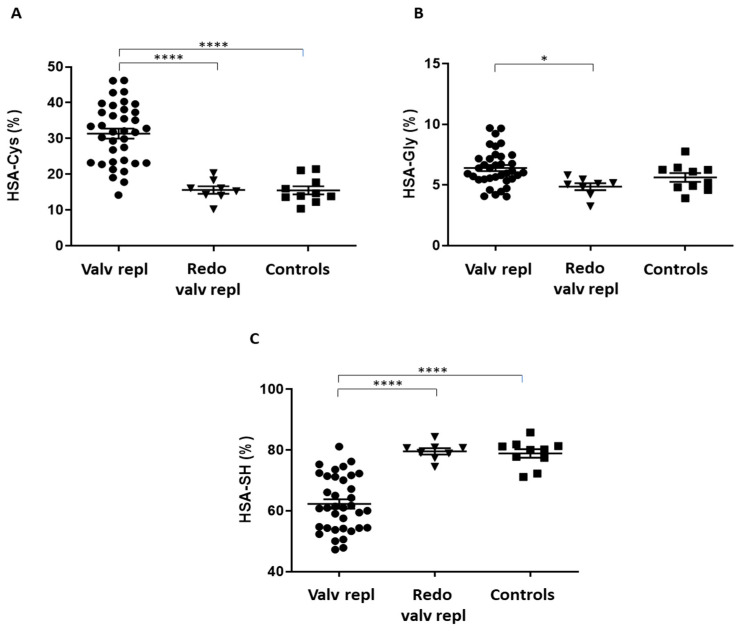
HSA proteoforms in patients and control subjects. Percentages of (**A**) HSA-Cys, (**B**) HSA-Gly, (**C**) HSA-SH were measured in serum obtained from patients with valvular disease before valve replacement (Valv repl, *n* = 36), patients requiring a second valve replacement to a degenerated bioprosthetic valve (Redo valv repl, *n* = 8), and healthy donors (Controls *n* = 10) by LC–mass spectrometry. Data are expressed as mean ± SEM. * *p* < 0.05; **** *p* < 0.0001 by one-way ANOVA followed by Tukey’s post hoc test.

The analysis of the HSA proteoforms shows that patients undergoing the first valve replacement have higher levels of HSA-Cys, the oxidized form of HSA, compared to patients undergoing the second surgery due to a degenerated bioprosthetic valve, and controls (Table 2 and Figure 2A). In addition, patients undergoing the first valve replacement have significantly higher levels of HSA-Gly, a modified form of HSA, due to the non-enzymatic addition of reducing sugars and/or their reactive degradation products to the protein, than those of patients undergoing the second surgery and controls (Table 2 and Figure 2B). In contrast, a marked reduction in the native form HSA-SH in patients undergoing the first valve replacement compared to patients undergoing the second surgery and controls was also detected (Table 2 and Figure 2C). No statistical difference was observed in total HSA levels between the control subjects and patients.

### 3.3. Levels of Free Sulfhydryl Groups in Serum from Patients and Controls

**Figure 3:**
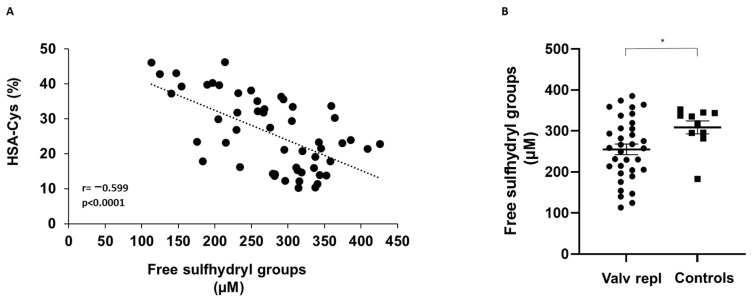
Correlation between HSA-Cys and free sulfhydryl groups and levels of free sulfhydryl groups in patients and control subjects. (**A**) HSA-Cys was measured in serum of all participants to the study by LC–mass spectrometry. Free sulfhydryl groups were detected in serum using Ellman’s reagent. (**B**) Free sulfhydryl groups detected in serum obtained from patients with valvular disease before valve replacement (Valv repl, *n* = 36), and healthy donors (Controls *n* = 10). Data are expressed as mean ± SEM. * *p* < 0.05.

Analysis of serum free sulfhydryl groups performed in the study population shows a negative correlation between the serum levels of sulfhydryl groups and HSA-Cys (Figure 3A). 

The levels of sulfhydryl groups in patients with aortic valve stenosis before the first surgical valve replacement were significantly lower compared to control subjects (Figure 3B). 

In contrast, the levels of sulfhydryl groups in patients requiring a second valve replacement due to a degenerated bioprosthetic valve were similar to those detected in control subjects (299 ± 12 µM and 309 ± 16 µM, for patients and controls respectively; *p* = 0.105). 

### 3.4. Aspirin Increases the Levels of Free Sulfhydryl Groups

**Figure 4:**
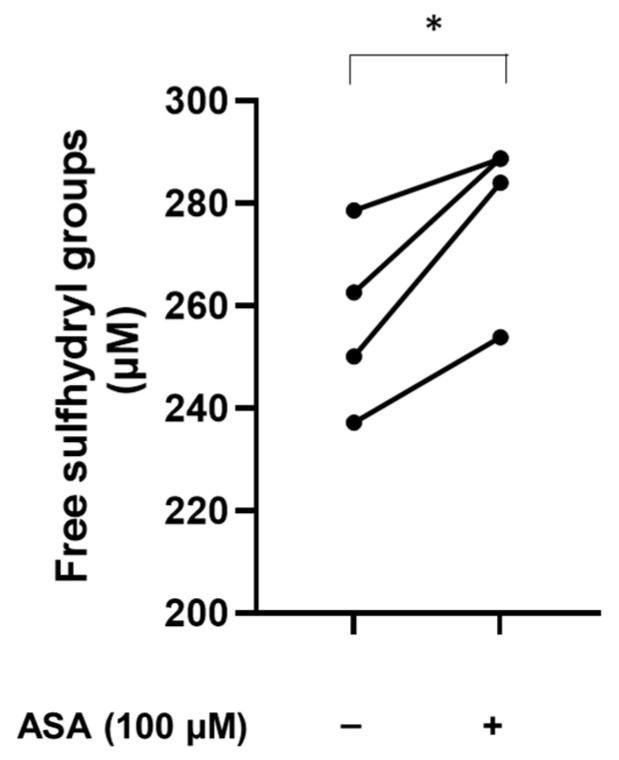
Analysis of free sulfhydryl groups in plasma treated with ASA. Blood obtained from healthy donors was incubated in vitro at 37 °C for 120 min in the presence or the absence of ASA. After centrifugation, plasma was collected, and free sulfhydryl groups were measured using Ellman’s reagent. *n* = 4. * *p* < 0.05 by paired Student’s *t*-test.

To assess whether ASA can affect the levels of free sulfhydryl groups in plasma proteins, blood obtained from healthy donors was treated in vitro with or without 100 µM ASA. As shown in Figure 4, sulfhydryl groups show a marked increase in plasma proteins after in vitro treatment with ASA, suggesting that the pharmacological treatment with ASA can affect the levels of free sulfhydryl groups and the oxidation status of plasma proteins.

### 3.5. N-Acetylcysteine and N-Acetylcysteine Amide (AD4/NACA) Reduce the Serum Levels of Cysteinylated Proteoform of Albumin 

**Figure 5:**
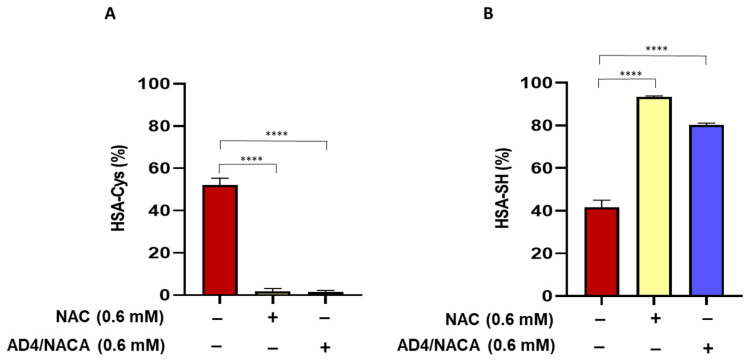
HSA proteoforms in serum of patients with aortic valve stenosis after in vitro treatment with NAC or its amide derivative AD4/NACA. Serum obtained from patients before valve replacement was incubated at 37 °C for 60 min in the presence of NAC or AD4/NACA. Levels of (**A**) HSA-Cys and (**B**) HSA-SH were measured by LC–mass spectrometry. *n* = 6. **** *p* < 0.0001 by one-way ANOVA followed by Dunnett’s post hoc test.

The serum obtained from patients with aortic valve stenosis before valve replacement was treated in vitro with NAC (0.6 mM) or AD4/NACA (0.6 mM). Results showed a marked reduction in the levels of the HSA-Cys proteoform, associated with a significant increase in the levels of HSA-SH (Figure 5A,B).

### 3.6. N-Acetylcysteine and N-Acetylcysteine Amide (AD4/NACA) Increase the Levels of Free Sulfhydryl Groups in Serum 

**Figure 6:**
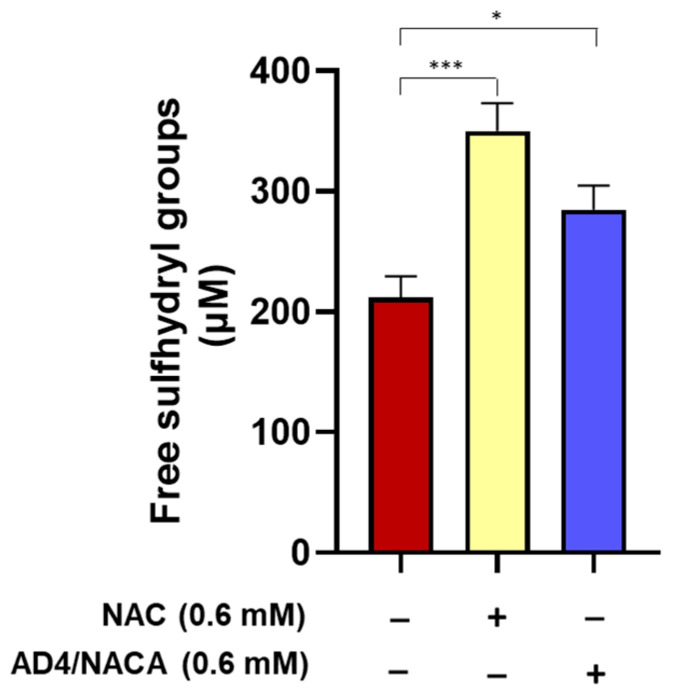
Analysis of serum free sulfhydryl groups after in vitro treatment with NAC or its amide derivative AD4/NACA. Serum of patients before valve replacement was incubated at 37 °C for 60 min in the presence of NAC or AD4/NACA. Free sulfhydryl groups were measured using Ellman’s reagent. *n* = 6. * *p* < 0.05; *** *p* < 0.001 by ANOVA followed by Dunnett’s post hoc test.

The serum samples obtained from patients with aortic valve stenosis before valve replacement was treated in vitro with NAC (0.6 mM) or AD4/NACA (0.6 mM) and subsequently analyzed for the measurement of the free sulfhydryl groups using Ellman’s reagent. A significant increase was detected with both these compounds, suggesting their ability to reduce oxidative stress and maintain redox homeostasis through the regeneration of free sulfhydryl groups of serum proteins (Figure 6).

### 3.7. N-Acetylcysteine and N-Acetylcysteine Amide (AD4/NACA) Increase the Total Antioxidant Activity of Serum from Patients

**Figure 7:**
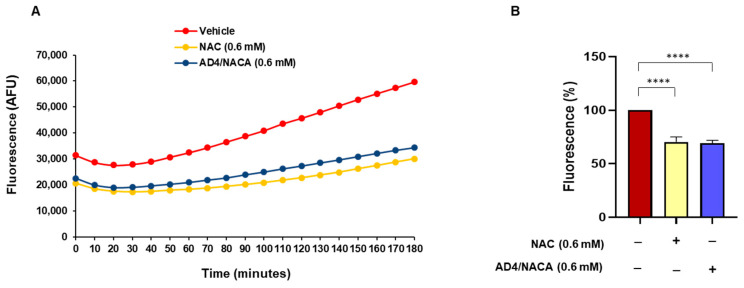
Antioxidant activity of serum after in vitro treatment with NAC or its amide derivative AD4/NACA. The serum of patients before valve replacement was incubated at 37 °C for 60 min in the presence of NAC or AD4/NACA. Antioxidant activity was detected by DCF fluorescence. (**A**) A representative curve of fluorescence of DCF (λex = 485 nm, λem = 532 nm) in the presence of AAPH as a generator of radical species. Data are expressed as arbitrary units of fluorescence (AFU) of DCF. (**B**) Effect of NAC and AD4/NACA on the oxidation of DCFH. The fluorescence of DCF was measured after 180 min after the start of the reaction. Data are expressed as % of fluorescence. *n* = 6. **** *p* < 0.0001 by ANOVA followed by Dunnett’s post hoc test.

The antioxidant activity was evaluated in serum samples of patients before the first valve replacement treated in vitro with NAC or AD4/NACA. After the addition of the radical generator AAPH, a lag time of ~50 min was detected. Afterward, the propagation phase started, and the radical generation was monitored for up to 180 min. In vehicle-treated samples, a gradual increase in radical generation was detected. In contrast, the treatment with NAC or AD4/NACA markedly prevented this radical generation (Figure 7A,B).

## 4. Discussion

In this paper, we showed for the first time that patients with severe aortic valve stenosis present high levels of serum HSA-Cys, the oxidized form of HSA, with a dramatic decrease in the native reduced proteoform HSA-SH compared to control subjects and patients undergoing a second valve substitution due to a degenerated bioprosthetic valve. Furthermore, the increase in HSA-Cys was paralleled by significant impairment of the total serum antioxidant activity and a decrease in the levels of free sulfhydryl groups, an index of the antioxidant capacity. Indeed, total sulfhydryl groups, and especially protein sulfhydryl groups, are considered to be the main circulating antioxidant defense, and an imbalance between the free sulfhydryl groups and disulfides contributes to the pathogenesis of several oxidative stress-mediated diseases [23]. In particular, free sulfhydryl groups are mainly found in HSA, the most abundant circulating protein, giving it important antioxidant properties, so that changes in the redox status of HSA are associated with an alteration in its beneficial antioxidant properties [24]. Of interest, in its oxidized form, circulating HSA may act as a carrier of low molecular weight thiols, which could be released into the subendothelial space to aggravate the oxidative stress of the diseased tissue [25].

**Figure 8:**
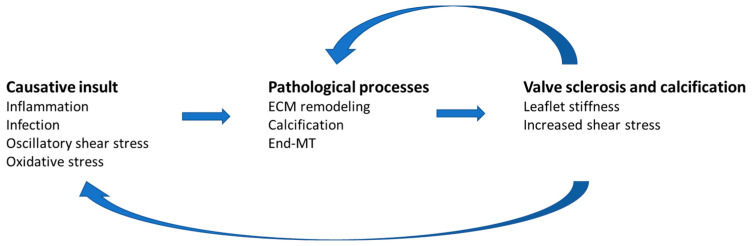
Feedback mechanisms that might perpetuate oxidative stress in patients with valvular disease. After the initiation of valvular inflammation and stenosis, mechanosensitive feedback pathways may provide the coupling link that promotes further valvular interstitial cell differentiation, valvular inflammation, and oxidative stress.

We also found that the levels of HSA-Cys in patients undergoing a second surgery due to a degenerated bioprosthetic valve are similar to control subjects. Thus, we hypothesize that the removal of the pathological valve is expected to stop the feedback maladaptive process, which includes oxidative stress. The increase in the cysteinylation of serum HSA reflects a condition of systemic oxidative stress, which could be amplified by mechanosensitive signaling pathways that convert mechanical forces experienced by valve leaflets and circulating cells into biochemical signals, thus providing a positive feedback loop that accelerates the progression of valvular disease in the advanced stages [26,27,28,29]. Indeed, it has been demonstrated that the pathological stretching of the valve tissue promotes maladaptive tissue remodeling by activating cell signaling, which adversely impacts the valve tissue properties, feeding back to further deteriorate valve function and propagate valve cell pathological responses [30]. In this process, the mechanosensitive pathways lead to the activation of circulating monocytes and platelets, inducing endothelial-mesenchymal transition, upregulation of proinflammatory and oxidant pathways, and promoting the valvular interstitial cells differentiation (Figure 8) [31,32]. These observations suggest a positive feedback mechanism by which the increased leaflet stiffness and altered shear stress conditions of early calcific aortic valve disease perpetuate and accelerate inflammation and oxidative stress, leading to the progression of valvular sclerosis and calcification [26,33,34]. 

Indeed, in a recent cohort study that evaluated systemic lipid peroxidation and the oxidative modifications of plasma proteins by measuring 2,4-dinitrophenylhydrazine, elevated oxidative stress was observed to correlate with the severity of aortic stenosis [35]. Levels of oxidative stress are also correlated with impaired systemic fibrinolysis, suggesting that the oxidative stress in calcific aortic valve disease patients might not simply reflect a localized phenomenon [35]. Results obtained from animal models also support a putative role for systemic inflammation in calcific aortic valve disease [36].

In this context, it is not surprising that valve replacement led to a decrease in cysteinylation of HSA to normal levels. Similarly, high levels of circulating inflammatory mediators associated with severe aortic stenosis are reduced with valve replacement. Indeed, transcatheter aortic valve replacement improves circulating protein composition to reverse myofibroblast activation in fibrotic valves and cardiac tissues, as shown in a proteomic analysis of patient sera [37]. Moreover, at 3 months after TAVR and 6 months post-surgical aortic valve replacement, there are significant reductions in circulating intermediate phenotype monocytes, which, being recruited at a later stage of inflammation, have a predominant role in inducing T-cell proliferation [38].

Furthermore, in accordance with the current guidelines, all our patients who have undergone a first valve replacement were in pharmacological treatment with ASA [39]. Therefore, we cannot exclude that ASA treatment, by regenerating the free sulfhydryl groups, contributes to a reduction in the levels of HSA-Cys. Indeed, besides its antiplatelet activity, the treatment with ASA is associated with a significant reduction in cardiovascular events and a slowdown in the progression of atherosclerosis [40]. In addition, it has been reported that ASA exhibits radical scavenging and antioxidant properties [41,42,43,44]. In particular, ASA administration protects low-density lipoproteins from oxidative modifications [45], the vascular wall from oxygen radical damage [46], and prevents protein oxidation by acetylation of amino groups of lysine residues [47] or scavenging hydroxyl radicals [48]. In addition, ASA reduced ROS production and oxidative stress in low-density lipoproteins-stimulated endothelial cells by downregulation of Nox4 and inducible nitric oxide synthase [49]. More recently, ASA administered for two months to healthy subjects significantly reduced the total oxidative status and the levels of oxidized low-density lipoproteins [50]. Several antioxidant effects of ASA may be attributed to an activation of gene transcription. For example, ASA increases the expression and the activity of heme-oxygenase-1, an important protein involved in the defense against oxidative tissue injury by several mechanisms [51]. In accordance with this, the enhanced heme-oxygenase-1 activity induced by ASA has also been documented in preclinical studies [44]. Additionally, ASA exerts antioxidant properties through the inhibition of NF-kB transcription factor, thus reducing ROS levels [41,52,53]. Of note, an in vivo study has shown that the treatment of healthy subjects with enteric-coated ASA for two weeks significantly increased the antioxidant capacity of plasma [54] and treatment with 160 mg of ASA until one day before the surgery markedly reduced the oxidative stress in patients scheduled for coronary heart bypass graft [55]. All these heterogeneous antioxidant properties of ASA are evidenced in several in vitro and in vivo studies and suggest that it plays a role at different levels. 

Finally, we demonstrated, at least in vitro, that the antioxidant compounds NAC and AD4/NACA [56,57] completely regenerate HSA-Cys to native reduced HSA, thus confirming our previous findings showing that albumin Cys34 can be regenerated in vitro by the dietary supplement NAC through a thiol–disulfide breaking mechanism [58,59], which leads to a full recovery of the HSA antioxidant [19,58]. The regenerative effects of NAC were subsequently verified in ex vivo conditions following both oral and intravenous administration [59]. A single-blinded, placebo-controlled crossover study was conducted on hypertensive individuals who were randomized into two groups. Participants received either NAC (600 mg/day) or a placebo, administered orally and intravenously, which both resulted in a significant decrease in HSA-Cys levels compared to the initial conditions (T0), with the maximal effect observed after 60 min. This reduction in HSA-Cys was accompanied by a simultaneous increase in native HSA-SH levels. Additionally, the total antioxidant activity of plasma significantly increased following NAC infusion compared to the placebo, suggesting that the restoration of Cys34 content may modulate oxidative stress in vivo and potentially have implications in diseases related to oxidative damage [59].

We acknowledge the limitations of this study. The results should be considered as preliminary due to the small size of the subject cohort and require validation with a larger sample size. Additionally, due to the limited sample size, we cannot completely rule out the impact of ongoing therapies. Lastly, the data is limited to subjects attending the local clinic, thus representing a specific geographical area.

## 5. Conclusions

This study contributes to highlighting the potential role of HSA oxidative modifications as a marker of oxidative stress in patients with aortic valve stenosis, a multifactorial process, including chemical, mechanical, and immunological factors. Although the contribution of each mechanism remains poorly understood, it represents an active field of investigation [32,60,61]. In this study, we showed that patients with a severe valvular disease that required surgical intervention with replacement of the aortic valve had higher oxidative stress compared to control subjects evidenced by an increase in HSA-Cys, the oxidized form of HSA, and a concomitant decrease in the reduced form (HSA-SH). Moreover, this group of patients showed a significant reduction in the free sulfhydryl groups in serum, an index of the antioxidant capacity of serum. Furthermore, patients who have already undergone valve replacement and are waiting for a second substitution due to a degenerated bioprosthetic valve had an oxidative stress status similar to control subjects, with similar HSA proteoform frequency and levels of free sulfhydryl groups in serum. As this second group of patients was in pharmacological treatment with ASA, an antithrombotic drug with antioxidant properties, we cannot exclude an additional contribution of ASA in the reduction in oxidative stress by regenerating the free sulfhydryl groups, which likely led to the decrease in HSA-Cys. We therefore found for the first time that HSA-Cys is increased in aortic valve stenosis representing both a stable and precise marker of oxidative stress as well as a therapeutic marker. Indeed, we show that HSA-Cys can be regenerated to native HSA by NAC and AD4/NACA, which fully replenishes its antioxidant activity, thus representing a potential treatment to reduce the oxidative stress burden in valvular disease. As well as being a biomarker of oxidative stress, HSA-SH also functions in the redox control of extracellular spaces and oxidative-based disease onset. Oxidative stress is a key driver of these processes, and inhibiting ROS is an important avenue to explore new therapies. Indeed, aortic valve stenosis can be potentially treated by targeting oxidative stress, which is a promising therapeutic approach. However, while animal and in vitro studies have shown some success in reducing oxidative stress in the context of aortic valve stenosis, clinical trials have not yet yielded similar benefits [6]. So far, the regeneration of the functional HSA-SH as a therapeutic target could represent a promising approach in oxidative-based diseases, including aortic valve stenosis.

## Figures and Tables

**Table 1:** Characteristics of patients enrolled in the study.

	Aortic Valve Replacement	Redo Aortic Valve Replacement	*p*
N	36	8	/
Male gender	18 (50.0)	5 (62.5)	0.701
Age (years)	78.0 (8.5)	70.5 (14)	0.100
Weight (Kg)	75.5 (12.5)	70.0 (43)	0.604
BMI (Kg/m^2^)	27.5 (3.7)	29.2 (12.2)	0.784
Hemoglobin (g/dL)	12.7 (2.1)	12.8 (3.8)	0.346
Platelets (×10^9^/L)	196 (80)	157.5 (89)	0.041
Neutrophils (×10^9^/L)	/	6.4 (53.8)	/
Lymphocytes (×10^9^/L)	/	2.0 (17.6)	/
Creatinine (mg/dL)	0.91 (0.40)	1.04 (0.36)	0.640
LDL (mg/dL)	71 (38)	92.5 (40)	0.439
Hypertension (*n*, %)	33 (91.7)	6 (75.0)	0.219
Dyslipidemia (*n*, %)	27 (75.0)	5 (62.5)	0.663
Diabetes (*n*, %)	7 (19.4)	2 (25.0)	0.659
Coronary artery disease (*n*, %)	6 (16.7)	1 (12.5)	1.000
LVEF (%)	61 (11.0)	55 (32.0)	0.692
Peak aortic gradient (mmHg)	72 (32)	75 (27)	0.737
Mean aortic gradient (mmHg)	43 (15)	48 (25)	0.608
Previous coronary artery bypass graft (*n*, %)	1 (2.8)	1 (12.5)	0.334
Oncological history (*n*, %)	3 (8.3)	1 (12.5)	1.000
Novel oral anticoagulants (*n*, %)	6 (16.7)	0 (0.0)	0.053
Statins (*n*, %)	21 (58.3)	4 (50.0)	0.710
Antidiabetic drugs (*n*, %)	7 (19.4)	2 (25.0)	0.659
Antihypertensive drugs (*n*, %)	32 (88.9)	5 (63.0)	0.100
Years since first surgery	/	8.7 (6.1)	/

Continuous variables were reported as median and IQR, and categorical variables were reported as absolute number (*n*) and frequency (%). *p* value was calculated using the Mann–Whitney test for continuous variables, and the chi-squared test or Fisher exact test as appropriate for categorical variables.

**Table 2:** HSA proteoforms distribution.

Albumin Proteoforms (%)	Aortic Valve Replacement	Redo Aortic Valve Replacement	Controls	*p* Value Valv Repl vs. Redo Valv Repl	*p* Value Valv Repl vs. Controls
*n*	36	8	10	/	/
% Cys (PA)	32.0 (14.4)	15.7 (3.1)	14.3 (4.2)	<0.001	<0.0001
% Gly (PA)	6.1 (1.8)	5.1 (0.8)	5.6 (1.5)	0.012	0.136
% SH (PA)	61.0 (17.0)	79.9 (4.0)	80.1 (3.9)	<0.001	<0.0001
HSA total (PA)	6.58 × 10^9^ (26.2 × 10^9^)	4.54 × 10^9^ (3 × 10^9^)	6.0 × 10^9^ (18.3 × 10^9^)	0.331	0.564

Data are expressed as median and IQR of PA (peak area).

## Data Availability

Data collected in the study will be made available using the data repository Zenodo (https://zenodo.org, accessed on 23 November 2023) with restricted access upon request to direzione.scientifica@ccfm.it. Any remaining information can be obtained from the corresponding author upon reasonable request.

## Supplementary Materials

The following supporting information can be downloaded at: https://www.mdpi.com/article/10.3390/biom13121713/s1, Figure S1: Levels of HSA-Cys in serum and plasma of controls and valvular disease patients.
