# Supplementary figures and images for: Albumin Thiolation and Oxidative Stress Status in Patients with Aortic Valve Stenosis
Source: Biomolecules. 2023 Nov 28;13(12):1713. doi: 10.3390/biom13121713 (PMC10742097; doi:10.3390/biom13121713)

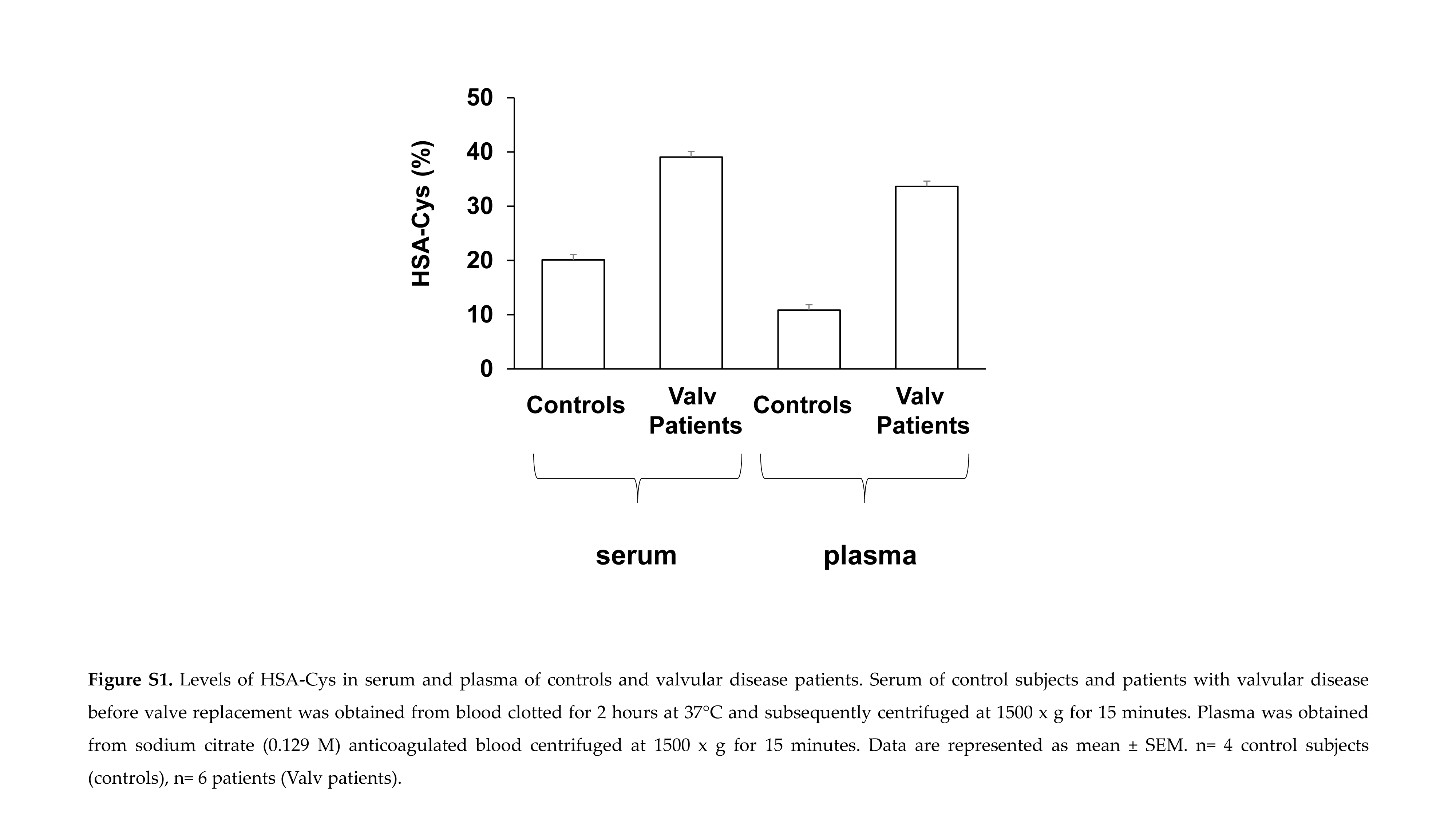

Supplement: Supplementary file 1 [file biomolecules-13-01713-s001.zip › biomolecules-2661926-supplementary.tif]
